# Mapping the Scientific Research on Nutrition and Mental Health: A Bibliometric Analysis

**DOI:** 10.3390/nu17030399

**Published:** 2025-01-22

**Authors:** Ramona Hiltensperger, Jennifer Neher, Lea Böhm, Annabel Sandra Mueller-Stierlin

**Affiliations:** Institute of Epidemiology and Medical Biometry, Ulm University, 89075 Ulm, Germany

**Keywords:** nutrition, mental health, psychiatry, bibliometric analysis

## Abstract

Background/Objectives: The connection between nutrition and mental health has become a point of growing interest. In response, nutritional psychiatry research has emerged as a field dedicated to understanding these interactions. A bibliometric analysis was conducted to map the growth and trends in this area, providing insights into its evolving scope and key research contributions. Methods: A literature search was conducted within the Web of Science Core Collection. Data analysis was performed using the result analysis and citation report options of Web of Science as well as the visualization software VOSviewer (Version 1.6.20). Results: A total of 31,556 articles on nutrition and mental health were published from 2000 to 2024 across various WoS categories, mainly in “Psychiatry”, “Public, Environmental & Occupational Health”, and “Nutrition & Dietetics”. The most prolific research groups are based in North America. Recent publications mainly cover seven clusters: (1) public health and social factors, (2) interventions and biological mechanisms, (3) the health of the elderly, (4) nutrition-related illnesses, (5) lifestyle behavior, (6) observational studies, and (7) pregnancy and the postpartum period. Conclusions: In view of the increasing research activities and growing allocation of resources in nutritional psychiatry research, it is important to define research priorities in close collaboration with service users and stakeholders. Research should be approached in a holistic manner through multidisciplinary research consortia to ensure comprehensive and impactful outcomes.

## 1. Introduction

The concept of “food is medicine” is a growing movement that emphasizes the idea that the food we eat serves not only as nourishment but also as a tool for preventing and treating diseases, improving health, and promoting overall well-being. This concept is rooted in the belief that whole, nutrient-dense foods can have therapeutic effects on the body and mind, much like conventional medicine, but with fewer side effects and long-term benefits. Hippocrates (c. 460–c. 370 BCE), the “Father of Medicine,” is often credited with popularizing the idea that food plays a central role in health. One of his famous quotes is “Let food be thy medicine and medicine be thy food”, reflecting his belief in the power of food to prevent and treat illness.

Also in modern medicine, diet is considered a key factor in managing conditions like digestive disorders, inflammation, and chronic diseases. Research linking diet to physical health conditions led to the recognition of nutrition as a vital part of medical treatment. Today, nutritional therapy is a core component of treating many physical health conditions like cardiovascular diseases, obesity, and metabolic disorders [1,2].

In contrast, in mainstream mental health care, nutritional approaches were slower to be implemented as a core component. Historically, mental health treatment focused more on psychological or pharmacological interventions rather than dietary changes. This may also be due to the mind–body dualism established by philosopher René Descartes in the 17th century that led to the long-standing separation between physical and mental health care [3]. This separation persists in many health care systems today.

Although the determinants of mental health are complex, the emerging evidence for nutrition as a crucial factor in the high prevalence and incidence of mental disorders is compelling. This suggests that diet is as important to psychiatry as it is to cardiology, endocrinology, and gastroenterology. Therefore, in 2015, the newly founded International Society for nutritional psychiatry research postulated “Nutritional medicine as mainstream in psychiatry” [4].

Since then, the number of publications on nutritional psychiatry has grown significantly. Now, approximately 10 years later, this rapidly expanding body of literature highlights the need for a comprehensive overview by examining the development of research topics, journals, authors, institutions, countries, and collaborations. For this purpose, a bibliometric analysis was conducted. Bibliometric analyses are a quantitative research method used to assess the patterns, trends, and impact of scientific publications in a specific field. Unlike systematic reviews, which typically involve a detailed data screening process to exclude irrelevant or low-quality studies, bibliometric analyses focus on examining large sets of publications based on predefined search terms. Herein, statistical methods are adopted to demonstrate knowledge structures and dynamic evolutions of a specific research field and reveal the number of publications and citations, keywords, themes, and patterns of collaboration in nutritional psychiatry research.

## 2. Materials and Methods

A literature search was conducted within the Web of Science Core Collection (WoSCC), a highly regarded, multidisciplinary research database maintained by Clarivate Analytics. To reveal the knowledge dynamics in nutritional psychiatry research, data analysis tools and the result analysis and citation report options of Web of Science as well as the visualization software VOSviewer (Version 1.6.20) were used.

### 2.1. Data Collection and Search Strategy

On 7th January 2025, we retrieved and downloaded data from the Web of Science Core Collection (WoSCC) database. In the WoSCC database, we searched articles using the search strategy “(nutrition* OR food* OR diet*) (Topic) and (psychiatr* or mental) (Topic) and 2000–2024 (year published)” for this bibliometric analysis.

### 2.2. Data Analysis

Within WoSCC, we analyzed the number of annual publications, the top 10 WoS categories, and the top 10 journals according to the number of retrieved publications. In addition, we analyzed the top 10 co-authors, institutions, and countries/regions publishing on nutrition and mental health. In terms of the subject areas covered, the top 10 meso- and micro-citation topics, the ten most frequently cited articles, and the five most cited publications on randomized controlled trials were analyzed.

Subsequently, we manually harmonized the spelling of keywords (e.g., “physical activity” = “physical-activity”) and imported the basic information of the identified literature from 2014 and from 2024 into VOSviewer for further visual analysis. For the analysis of collaboration networks (for researchers and countries), the minimum number of publications per author in 2014 and 2024 and per country to be included in the network analysis was set to two, three, or five, respectively. The minimum number of publications per keyword to be included in the network analysis was set to 10 or 20 for 2014 and 2024, respectively. For all other variables, default options were used for analysis.

## 3. Results

From 2000 to 2024, a total of 31,556 documents were retrieved in the Web of Science (WoS) Core Collection database, including 25,288 original research articles (80.1%) and 5095 review articles (16.1%). Almost all articles (*n* = 30,347, 96.2%) were published in English.

### 3.1. Publications 

#### 3.1.1. Annual Publication Trends

**Figure 1:**
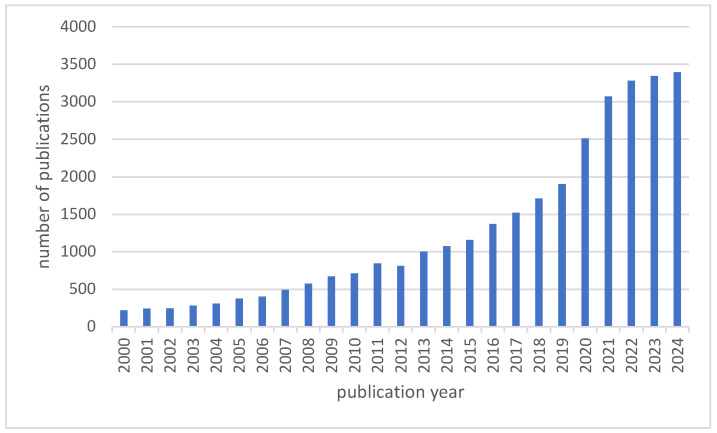
The number of annual publications on nutrition and mental health.

The number of articles published is increasing year by year (minimum: 2000 (*n* = 222); maximum: 2024 (*n* = 3392)), with a particularly strong increase of 61% between 2019 (*n* = 1905) and 2021 (*n* = 3071), as seen in Figure 1.

**Figure 2:**
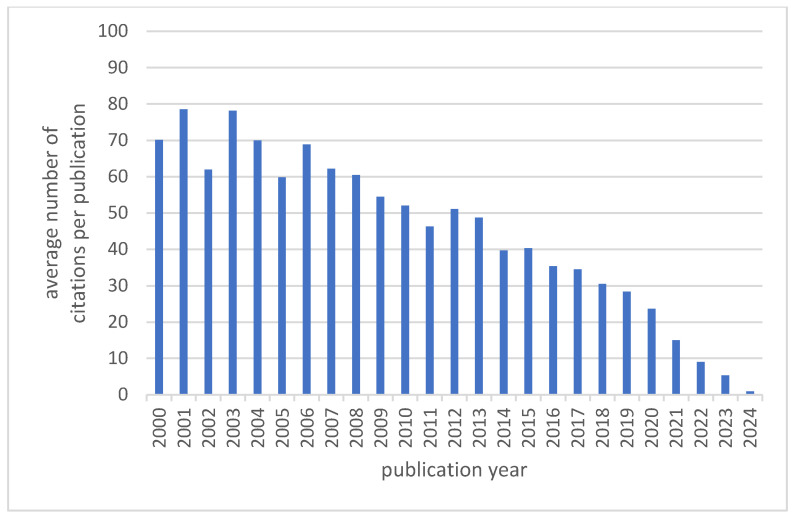
The average number of citations per annual publications on nutrition and mental health.

Citation reports for annual publications reveal a total of 866,657 citations for the 31,556 included publications. This corresponds to an average of 27.5 citations per publication, with a decreasing average number of citations per publication over time (see Figure 2). Most citations are reported for publications from 2020 (*n* = 59,512).

#### 3.1.2. Categories in Web of Science

The analysis of subject categories in the source reveals a broad distribution of Web of Science (WoS) categories (see Table 1). Half of the top 10 most prominent WoS categories in nutritional psychiatry research are medical in nature, accounting for 41.0% of the total publications (*n* = 12,939) across categories such as “Psychiatry”, “Medicine General Internal”, “Pharmacology Pharmacy”, “Clinical Neurology”, “Psychology Clinical”, and “Pediatrics”. Nearly one-eighth of the publications was classified under the WoS category “Nutrition Dietetics” (*n* = 4257, 13.5%).

#### 3.1.3. Journals

Nutritional psychiatry research has been published in 6323 journals (see Table 2). The journal with the most publications on this topic is *Nutrients* (*n* = 744, 2.4%), followed by *PLOS ONE* (*n* = 502, 1.6%), the *International Journal of Environmental Research and Public Health* (*n* = 420, 1.3%), and the *Journal of Affective Disorders* (*n* = 361, 1.1%).

### 3.2. Actors

#### 3.2.1. Authors

Four of the top ten most prolific authors—Verhagen Hans (*n* = 46), Martin Ambroise (*n* = 45), Marchelli Rosangela (*n* = 43), and Moseley Bevan (*n* = 42)—have collectively contributed to scientific opinions on the substantiation of health claims related to nutritional supplements and mental state and performance for the European Food Safety Authority (EFSA). To provide a clearer overview of the top 10 authors in the academic research landscape, these EFSA-related publications were excluded from the analysis (“exclude (EFSA) (Topic)”, *n* = 66). Among the remaining top ten authors, five primarily published in the WoS category “Psychiatry”, two in “Public Environmental Occupational Health” (focusing on food insecurity, especially in HIV patients and on pre- and postnatal mental health), and one each in the WoS categories “Nutrition Dietetics” (focusing on food insecurity, especially in HIV patients) and “Neurosciences” (focusing on the microbiota–gut–brain axis) (see Table 3).

**Figure 3:**
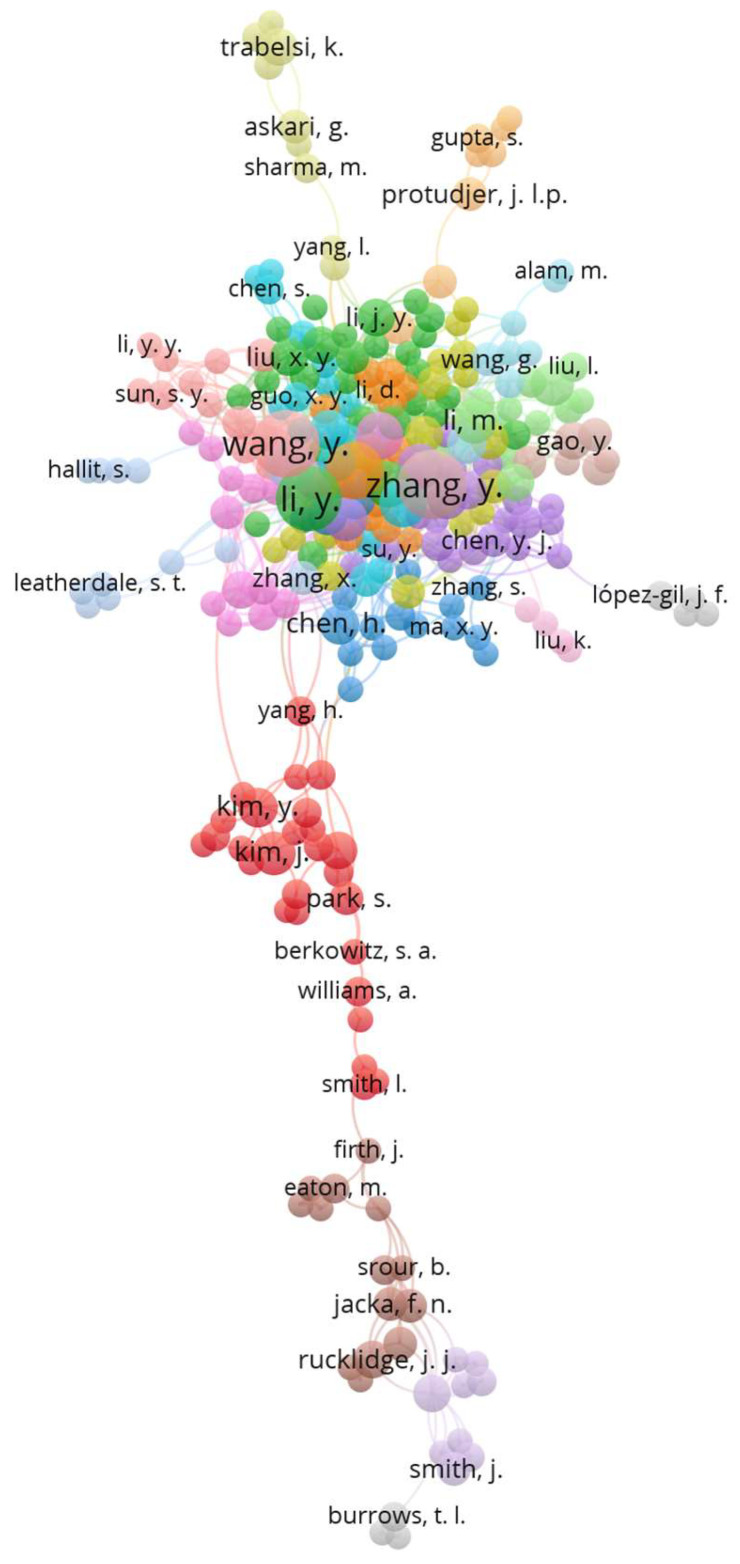
Top 263 most prolific co-authors in 2024 and their relationships within 20 clusters (indicated by different colors), created by VOSviewer.

The analysis of publications from 2024 identified a total of 18,437 authors. A subset of 447 authors, each with three or more publications, were classified as the most prolific ones. Network analysis using VOSviewer revealed 20 clusters among 263 of those authors (see Figure 3). The network has grown considerably since 2014, when it was formed from a network of just 17 authors with two or more publications (cluster 1: Anstey, KJ, Butterworth, P, Cherbuin, N; cluster 2: Allender S, Jacka FN, Logan, AC, Waters, E; cluster 3: Berk, M, O’Neill, A, Sarris, J, Schweitzer, I; cluster 4: Fuller-Tyszkiewicz, M, Hoare, E, Lewis, AJ, Millar, L, Skouteris, H, Swinburn, B).

#### 3.2.2. Institutions

The ten most prolific institutions, each with 433 or more publications on nutritional psychiatry research, include five institutions from the USA, four from Europe, and one from Canada (see Table 4). These institutions primarily focused on publications in the WoS category “Psychiatry” (eight institutions among the top ten). The most prolific institution, the *University of California System* in the USA, mainly published in the WoS category “Public Environmental Occupational Health”. The *Institut National de la Santé et de la Recherche Médicale (Inserm)* in France was the only institution with a primary focus on “Nutrition Dietetics”.

#### 3.2.3. Countries/Regions

The ten most prolific countries/regions in this subject area are in North America (*n* = 13,117, 41.6% from USA and Canada in total), in Asia/Oceania (*n* = 7358, 23.3% from Australia, the People’s Republic of China, Japan, and India in total) and in Europe (*n* = 6710, 21.3% from England, Italy, Germany, and Spain in total).

**Figure 4:**
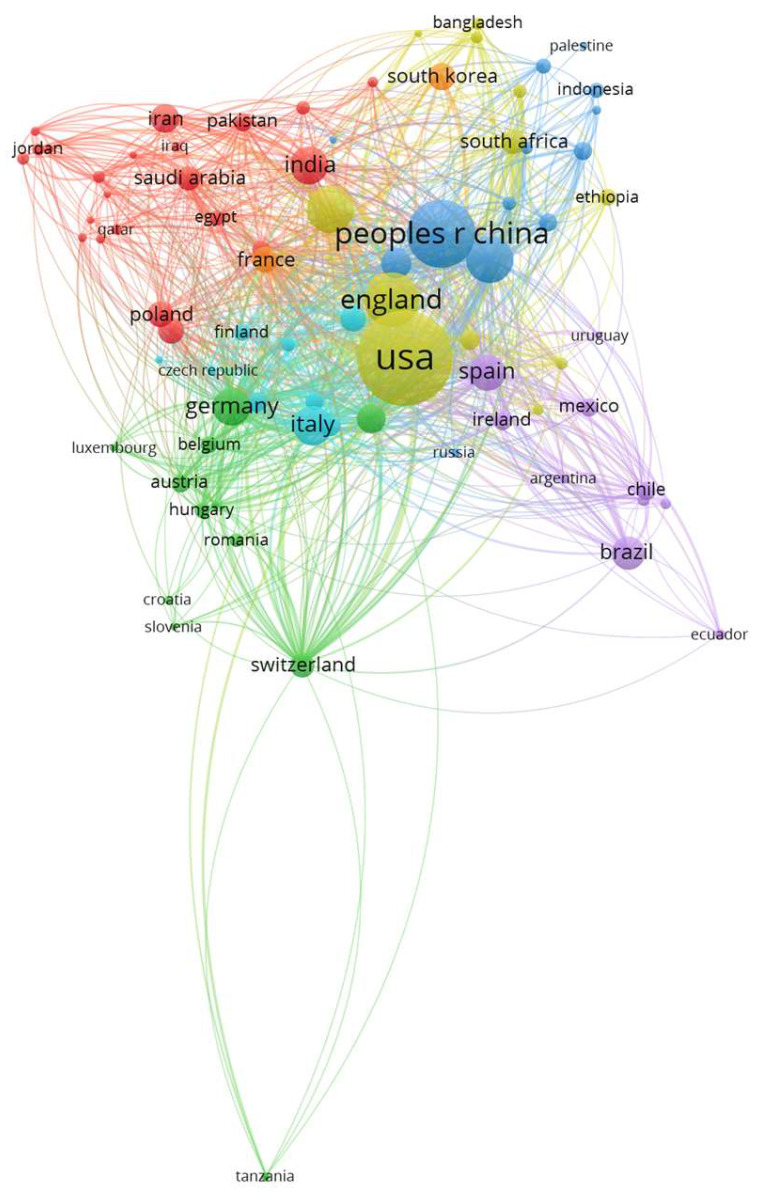
Co-authorship networks in 2024 by countries with seven clusters (indicated by different colors), created with VOSviewer.

Regarding the global distribution of co-author networks in 2024, seven clusters of countries/regions (with a minimum of five publications) were identified (see Figure 4): The first cluster (red) includes 22 Western Asian countries/regions, such as India, Saudi Arabia, and Iran. There are two clusters with European countries; the second one (green) consists of thirteen countries/regions and the third one (petrol) consists of nine countries. The fourth and fifth cluster (blue and yellow), the most prolific ones, span 13 or 12 countries/regions, including the USA, Canada, and England (fourth cluster) as well as the People’s Republic of China, Australia, and Japan (fifth cluster), but also low–middle-income countries such as South Africa or Ethiopia (fourth cluster) or Indonesia and Palestine (fifth cluster). The sixth cluster (purple) is the largest one in terms of geographical distance and includes a total of ten countries from South America and Europe. Only France and South Korea form the seventh cluster (orange).

### 3.3. Themes

#### 3.3.1. Keywords

**Figure 5:**
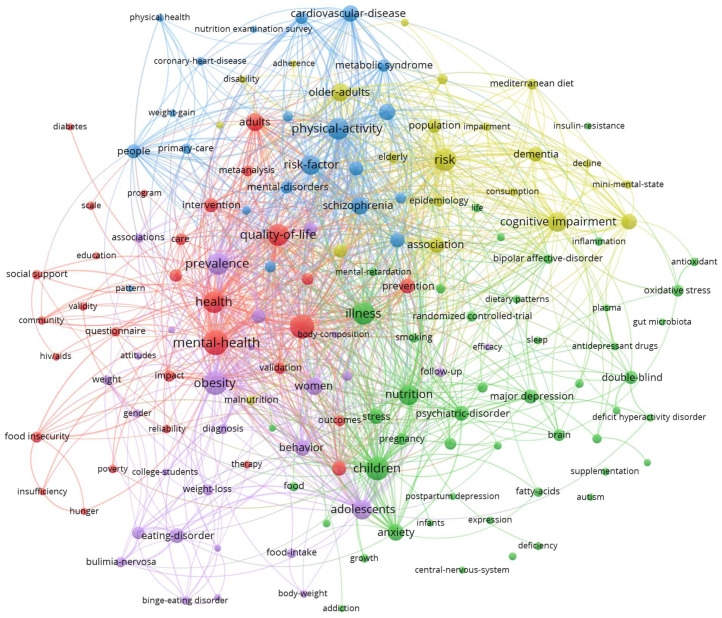
Keyword mapping for all articles published on nutrition and mental health in 2014 with five clusters (public health and social factors (red), interventions and biological mechanisms (green), health of the elderly (yellow), nutrition-related illnesses (purple), lifestyle behavior (blue)), created using VOSviewer.

**Figure 6:**
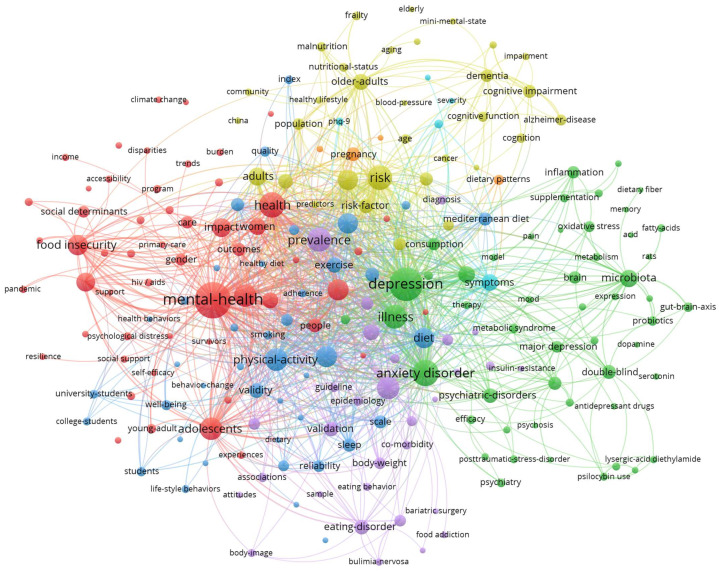
Keyword mapping for all articles published on nutrition and mental health in 2024 with seven clusters (public health and social factors (red), interventions and biological mechanisms (green), health of the elderly (yellow), nutrition-related illnesses (purple), lifestyle behavior (blue), observational studies (petrol), and pregnancy and postpartum period (orange)), created using VOSviewer.

Mapping the 11,670 keywords of the articles published in 2024 reveals seven clusters with 217 keywords (identified for at least 20 publications), which could be titled as follows: (1) public health and social issues (red, 54 keywords), (2) interventions and biological mechanisms (green, 51 keywords), (3) the health of the elderly (yellow, 32 keywords), (4) nutrition-related illnesses (purple, 30 keywords), (5) lifestyle behavior (blue, 41 keywords), (6) observational studies (petrol, 5 keywords), and (7) pregnancy and the postpartum period (orange, 4 items) (see Figure 5 and Figure 6).

A closer look at the keyword maps in in Figure 5 and Figure 6 (and Supplementary File S1) illustrates that nutrition and mental health in the context of the COVID-19 pandemic was of interest (see Figure 6, red cluster). Poverty and food insecurity, especially in low-income settings, were also frequently addressed (see Figure 6, red cluster), as already performed in 2014 (see Figure 5, red cluster). In comparison to 2014, health disparities, stigma, self-efficacy, and climate change are included in the keyword map (see Figure 6, red cluster). Visual analysis of the second cluster (green cluster) reveals that research focused on depression and anxiety, while schizophrenia was less addressed. In contrast, in 2014, schizophrenia, major depression, and anxiety seemed to be equally important. Moreover, attention deficit/hyperactivity disorder (see Figure 5, green cluster) vanished from the keyword map in 2024. The delivered nutrition-related interventions included supplementation, pre- and probiotics, and the ketogenic diet (see Figure 6, green cluster). The exploration of biological mechanisms, especially related to the gut microbiota but also related to inflammation or oxidative stress, seems to have been studied in 2024 (see Figure 6, green cluster), while the gut microbiota was rarely investigated in 2014. Also, the gut–brain axis has just emerged in the last few years (see Figure 6, green cluster). Notably, randomized controlled trials (see Figure 6, red cluster) were discussed much less frequently than meta-analyses (see Figure 6, green cluster). When it comes to the health of the elderly (see Figure 6, yellow cluster), malnutrition, frailty and cognitive impairment, dementia, and Alzheimer’s disease were revealed to be the most prominent topics. There were hardly any obvious changes to this subject area between 2014 (see Figure 5, yellow cluster) and 2024 (see Figure 6, yellow cluster). Interestingly, the Mediterranean diet was included in the third cluster in 2014 (see Figure 5, yellow cluster) but in the fourth cluster in 2024 (see Figure 6, purple cluster). The most researched nutrition-related illnesses in the mental health context were obesity and eating disorders (see Figure 6, purple cluster). However, given the size of the dots and the small number of ties, eating disorders tend to play an outsider role in the keyword maps from 2014 (see Figure 5, purple cluster) and 2024 (see Figure 6, purple cluster). The map from 2024 also includes metabolic syndrome (see Figure 6, green cluster) and comorbidities, such as diabetes mellitus and cardiometabolic health (see Figure 6, purple cluster). In 2014, dietary issues relating to somatic health like cardiovascular disease and diabetes were addressed, particularly in connection with physical activity and for the target group of people with schizophrenia (see Figure 5, blue cluster). Interestingly, the rather independent cluster from 2014 (see Figure 5, blue cluster) has tended to move to the center of the map in recent years and appears to form a second layer in 2024 (see Figure 6, blue cluster). This research addresses all lifestyle behaviors related to health promotion and stress reduction, such as physical activity, alcohol consumption, smoking, and sleep. Basic dietary recommendations to improve diet quality, e.g., by means of a Mediterranean diet pattern, are covered (see Figure 6, blue cluster). This probably indicates that lifestyle factors are currently relevant in all research clusters related to nutrition and mental health. In 2024, two new clusters emerged: the sixth one focuses on observational studies, such as the National Health and Nutrition Examination Survey (NHANES) evaluating cross-sectional associations between dietary patterns and mental health (see Figure 6, petrol cluster), while the seventh one focuses on diet in pregnancy and the postpartum period (see Figure 6, orange cluster).

Citation topics represent groups of papers that are related through citation links and are organized at both the meso and micro levels. The ten most significant meso- and micro-citation topics are listed in Table 5. These topics reveal that several professional disciplines at the macro level—such as “nutrition & dietetics”, “psychiatry”, “psychiatry & psychology”, and “obstetrics & gynecology”—are active in nutritional psychiatry research. Several health conditions are frequently explored in this context, including “neurodegenerative diseases”, “inflammatory bowel disease & infections”, and “substance abuse” at the macro level, and “eating disorders”, “sarcopenia”, “dementia”, “obesity”, and “postpartum depression” at the micro level. Commonly discussed risk factors and mechanisms include “vitamin metabolism” and “lipids” at the macro level, as well as “gut microbiota”, “docosahexaenoic acid”, “olanzapine”, “food insecurity”, and “physical activity” at the micro level.

#### 3.3.2. Most Cited Papers

The most frequently cited articles in nutritional psychiatry research are not highly specific studies but rather comprehensive works addressing the global burden of disease and the body–mind connection (see Table 6).

In a second step, we refined our search by including the search term “((randomized controlled trial) or RCT) (all fields)” and this results in 2336 hits (7.4%). The five most cited original research articles examine the effectiveness of nutrition-related interventions for elderly, vulnerable groups (living with cancer or being pregnant) and people living with major depression (see Table 7).

## 4. Discussion

In this bibliometric analysis, covering the years 2000 to 2024, a total of 31,556 documents related to nutrition and mental health were retrieved from the Web of Science (WoS) Core Collection. The number of publications has steadily increased year by year, with a 15-fold rise in the number of articles published between 2000 and 2024. These publications are distributed across various WoS categories, with a strong emphasis on medical disciplines such as “Psychiatry,” but also “Public, Environmental & Occupational Health” and “Nutrition & Dietetics”. The most prolific co-authors in this field predominantly publish within the “Psychiatry” category. While five of the top co-authors are based in Australia, five out of the top ten most prolific institutions are primarily located in the United States. Keyword analysis revealed seven distinct research clusters for the year 2024: (1) public health and social factors (including food insecurity and health disparities), (2) interventions and biological mechanisms, (3) the health of the elderly (including dementia, frailty, and malnutrition), (4) nutrition-related illnesses (including eating disorders and obesity), (5) lifestyle behavior (including physical activity and stress), (6) observational studies, and (7) pregnancy and the postpartum period. Among these, depression and anxiety disorder appear to be the most prominent focus in nutritional psychiatry research.

In view of the increasing research activities in this area, it is not surprising that the topic has gained growing interest from professional societies in mental health. However, it is striking that the latest guidelines make either no clear recommendations or only recommendations with a low level of evidence [20,21]. This can be attributed to the rarity and limited quality of systematic reviews on the effectiveness of dietary interventions for people living with severe mental illness [22,23,24]. Moreover, systematic reviews on the effectiveness of dietary interventions for people with major depressive disorder have shown significant heterogeneity and low quality of studies [25,26,27], limiting result generalizability. Regarding dietary interventions for people with schizophrenia, the authors of the EPA guidance identified only one review from 2016, which, in turn, found no studies meeting the inclusion criteria [28]. In line with our findings of this bibliometric analysis, this indicates a lack of randomized controlled trials and a loss of focus on the target group of people living with severe mental illness, such as schizophrenic spectrum disorders. Taken together, this highlights further research needs for higher-quality reviews, which could then serve as the basis for the next guidelines for further interventional research to establish stronger dietary recommendations for individuals with mental health conditions [21].

Furthermore, keyword mapping revealed the multifactorial nature of nutritional psychiatry, involving biological, psychological, and social factors and thereby emphasizing the importance of the Biopsychosocial and Lifestyle Model as outlined in the clinical practice guidelines for mood disorders of the Royal Australian and New Zealand College of Psychiatrists [29]. In line with this model, biological factors, such as genetics, medication, and co-occurring health conditions, must be considered in clinical practice and research. It is important to bear in mind that psychological challenges, like motivational issues, cognitive impairments, and symptom fluctuations, can affect adherence to dietary interventions. In addition, social and cultural influences, as well as economic barriers like food insecurity, further complicate implementation [30,31]. It is crucial to view nutrition through a biopsychosocial lens, recognizing that dietary interventions are not solely about nutrients but also about the broader psychological and social dimensions of health, and, thus, these three areas should not be considered separately in different research clusters but should be tackled simultaneously. Therefore, it is promising to see that a research cluster (fourth cluster on “lifestyle-behavior”, blue) has already emerged in nutritional psychiatry, which appears to be interwoven with a large number of different topics. To deal with this complexity in research, advanced statistical models must be applied to account for individual intervention uptake and other confounding factors. In addition, qualitative research is needed to explore service users’ and service providers’ experiences to better understand challenges in the sustainable implementation of dietary interventions in mental health settings. With the aim to improve the rigor and clinical relevance of future clinical trials in this respect, the International Society for Nutritional Psychiatry Research recently published methodological and reporting recommendations for clinical trials [32]. An emphasis was placed on the importance of a multidisciplinary research team and the integration of co-design processes into the design and conduct of clinical research in nutritional psychiatry. It should always be borne in mind that those with (severe) mental illness often represent a particularly vulnerable population. Ethical considerations must guide nutritional psychiatry research, ensuring that culturally appropriate, sustainable, and socially inclusive interventions and implementation strategies are applied.

Over the past years, important milestones through pioneering research projects have been set in this way by a group of Australian researchers who were identified as one of the most prolific ones in nutritional psychiatry research. For instance, the “Keeping the Body in Mind program” addresses the high rates of physical health issues in individuals with severe mental illnesses. It was first implemented in South Eastern Sydney Local Health District by Scott Teasdale, among others, and combines diet, physical activity, and psychoeducation to improve both mental and physical health outcomes [33,34]. The SMILES trial, led by Felice Jacka from the Food & Mood Centre at Deakin University, was a landmark randomized controlled trial that investigated the impact of a Mediterranean-style diet on the mental health of people with major depression [35]. Thereafter, the same research group conducted another trial—the CALM trial—that examined the effects of a modified Mediterranean diet on mental health outcomes in people with severe mental illness (i.e., having a diagnosis of schizophrenia, bipolar disorder, or major depressive disorder) [36]. As the samples in all studies were small and were confined to Australian populations, further such RCTs should now be undertaken across the globe [20].

The broad spectrum of intervention approaches, outcomes, and target groups identified by the analysis of meso- and micro-citation topics and keywords raises the question of which research questions should be prioritized first. Research efforts must focus on global health priorities, including addressing malnutrition and mental health disparities, as outlined in the WHO’s Sustainable Development Goals (SDGs). The emphasis should be on creating research frameworks that are adaptable to different contexts, ensuring that interventions are not only effective in high-income countries like Australia but also culturally and economically viable across diverse global settings. The close links between countries with different income levels, which presumably exist due to the historical research priorities in the area of malnutrition, can prove beneficial here. The potential for the sustainable improvement of health care services for people living with mental illnesses should be the prime consideration. In addition to the effectiveness of the intervention on the chosen outcome, the impact on the lives of the service users, the practicability and sustainability of such interventions, and equity considerations have to be taken into account. This can only be achieved if service users and stakeholders are involved in the planning of research projects from the initial stages.

Particularly in the case of intervention approaches that are based on a fundamental change in dietary habits and daily routines—such as a ketogenic diet—consideration must be given to the adverse effects these can have, especially on social life [37]. Particularly in this vulnerable target group, the high costs of such a diet, i.a., due to the emphasis on high-quality whole foods, are a concern. Additionally, the diet may lead to social exclusion, as its specific requirements may not be compatible with public meals or social gatherings. These financial and social challenges should be carefully considered. Now that the first randomized controlled trials on the effectiveness of ketogenic diets are already starting [38], qualitative research methods are urgently needed to explore the subjective expectations and experiences of the involved users and professional groups (as carried out in other target groups [39]).

In general, the subjective perspectives of people living with mental health conditions and those of stakeholders, as well as health economic considerations, are crucial for the development of guidelines on nutrition and mental health. These perspectives need to be included in the compilation of recommendations, in addition to proof of effectiveness in high-quality randomized controlled trials. Stakeholder involvement in nutrition psychiatry research should include the early engagement of diverse groups, such as service users, mental health professionals, nutrition professionals, and policymakers, from the initial stages of research design. This can be achieved through co-design workshops, focus groups, and regular feedback loops throughout the study. Additionally, involving stakeholders in data interpretation and dissemination will ensure that the research findings are both relevant and actionable in real-world settings. Multi-professional research consortia are needed to implement such research approaches. It is imperative that mental health researchers, nutrition scientists, and stakeholders are involved so that all areas of expertise are adequately represented. Other professional groups, e.g., social scientists and health economists, should be involved for pursuing specific research objectives in order to gather a holistic view on the research topic [40].

### Strengths and Limitations

The WoSCC serves as one of the most comprehensive and authoritative resources for academic research, providing access to a vast collection of peer-reviewed journals, conference proceedings, and other scholarly literature. The bibliometric analysis allowed us to rapidly understand the dynamics of knowledge pertaining to the research on nutrition and mental health. One limitation of this bibliometric analysis lies in the inclusion of studies that may not meet the precise thematic focus of the research area, particularly those that are peripheral or only indirectly related to the topic of nutrition and mental health. As bibliometric analyses typically do not incorporate strict data screening, some papers that are not directly relevant to the research question may be included in this analysis. This is a distinction from systematic reviews, which tend to apply more stringent inclusion criteria. While this method allows for a comprehensive exploration of trends within the broader literature, it may introduce some noise into the analysis. Another limitation of this project lies in the simple and selective search strategy. In our study, we have applied a whole-food approach to the topic of nutrition and mental health, focusing on research looking at the broader concept of diet and food patterns, rather than isolating specific nutrients (e.g., vitamins, minerals, or individual dietary components). By excluding nutrient-specific research terms, we aimed to capture a more holistic view of how dietary habits and food intake as a whole can influence mental health outcomes, rather than concentrating on isolated effects of individual nutrients. In addition, the keyword analysis was conducted pragmatically—without structured keyword assignment. Moreover, a sub-analysis according to research methodology (e.g., “randomized controlled trials” as available in PubMed) was not implementable.

## 5. Conclusions

This is the first bibliometric analysis that describes the scientific literature on nutrition and mental health. Our results reveal an extensive body of literature published in the field of nutritional psychiatry research, with a continuous increase in the annual scientific output. Future research should be considerate of global health priorities, inclusivity, and ethical considerations. In order to do justice to the complexity of the topic, multi-professional research groups that take diverse perspectives in the evaluation into account are necessary. This collaborative approach will foster a deeper understanding of the mind–body connection, ultimately leading to the recognition of nutrition as a foundational element of holistic health care, particularly for individuals living with mental health conditions.

## Figures and Tables

**Table 1:** Top 10 most important Web of Science categories on nutrition and mental health.

WoS Category (Number of Publications, %)	Main Journal, Publisher (Number of Publications, %)
Psychiatry (5087, 16.1%)	Journal of Affective disorders, Elsevier (266, 5.2%)
Public Environmental Occupational Health (4497, 14.3%)	International Journal of Environmental Research and Public Health, MDPI (420, 9.3%)
Nutrition Dietetics (4257, 13.5%)	Nutrients, MDPI (744, 17.5%)
Medicine General Internal (2394, 7.6%)	BMJ OPEN, BMJ (263, 11.0%)
Neurosciences (2219, 7.0%)	Nutritional Neuroscience, Taylor & Francis (114, 5.1%)
Pharmacology Pharmacy (1582, 5.0%)	Psychopharmacology, Springer Nature (65, 4.1%)
Clinical Neurology (1409, 4.5%)	Journal of Affective disorders, Elsevier (266, 18.9%)
Pediatrics (1321, 4.2%)	Pediatrics, Amer Acad Pediatrics (102, 7.7%)
Psychology Clinical (1146, 3.6%)	International Journal of Eating Disorders, Wiley (162, 14.1%)
Endocrinology Metabolism (1131, 3.6%)	Annals of nutrition and metabolism, Karger (48, 4.2%)

**Table 2:** Top 10 journals with most publications on nutrition and mental health.

Journal, Publisher	Number of Publications, %
*Nutrients*, MDPI	744, 2.4%
*PLOS ONE*, Public Library Science	502, 1.6%
*International Journal of Environmental Research and Public Health*, MDPI	420, 1.3%
*BMC Public Health*, Springer Nature	361, 1.1%
*Journal of Affective Disorders*, Elsevier	266, 0.8%
*BMJ OPEN*, BMJ	263, 0.8%
*Frontiers in Psychiatry*, Frontiers	212, 0.7%
*Appetite*, Elsevier	199, 0.6%
*Frontiers in Public Health*, Frontiers	196, 0.6%
*Cureus Journal of Medical Science*, Springer Nature	177, 0.6%

**Table 3:** Top 10 most prolific co-authors publishing on nutrition and mental health.

Name (Number of Publications, %)	Main WoS Category (Number of Publications, %)
Jacka, Felice N (110, 0.3%), Australia H-Index: 69	Psychiatry (46, 41.8%)
Berk, Michael (83, 0.3%), Australia H-Index: 125	Psychiatry (51, 61.4%)
Teasdale, Scott (53, 0.2%), Australia H-Index: 22	Psychiatry (27, 50.9%)
Frongillo, Edward A. (51, 0.1%), USA H-Index: 81	Nutrition Dietetics (16, 31.4%)
Weiser, Sheri D. (48, 0.1%), USA H-Index: 57	Public Environmental Occupational Health and Public Health (22, 45.8%)
Hay, Phillipa (43, 0.1%), Australia H-Index: 61	Psychiatry (18, 41.9%)
Cryan, John F. (43, 0.1%), Ireland H-Index: 133	Neurosciences (20, 46.5%)
Sarris, Jerome (39, 0.1%), Australia H-Index: 49	Psychiatry (25, 64.1%)
Stubbs, Brendon (39, 0.1%), England H-Index: 105	Psychiatry (29, 74.4%)
Tomlinson, Mark (37, 0.2%), South Africa H-Index: 64	Public Environmental Occupational Health and Public Health (43.2%)

**Table 4:** Top 10 most prolific institutions publishing on nutrition and mental health.

Name of Institution, Country (Number of Publications, %)	Main WoS Category (Number of Publications, %)
University of California System, USA (1129, 3.6%)	Public Environmental Occupational Health and Public Health (231, 20.5%)
University of London, England (1049, 3.3%)	Psychiatry (291, 27.7%)
Harvard University, USA (1002, 3.2%)	Psychiatry (248, 24.8%)
University of Toronto, Canada (582, 1.8%)	Psychiatry (157, 27.0%)
Johns Hopkins University, USA (530, 1.7%)	Psychiatry (128, 24.2%)
Harvard Medical School, USA (504, 1.6%)	Psychiatry (144, 28.6%)
University system of Ohio, USA (479, 1.5%)	Psychiatry (81, 16.9%)
Institut National de la Sante et de la Recherche Medicale Inserm, France (455, 1.4%)	Nutrition Dietetics (101, 22.2%)
University College London, England (434, 1.4%)	Psychiatry (99, 22.8%)
King’s College London, England (433, 1.4%)	Psychiatry (189, 43.6%)

**Table 5:** Top 10 meso- and micro-citation topics in nutritional psychiatry research.

Name of Meso-Citation Topic (Number of Publications, %)	Name of Micro-Citation Topic (Number of Publications, %)
Nutrition & Dietetics (7081, 22.4%)	Eating Disorders (2198, 7.0%)
Psychiatry (2523, 8.0%)	Obesity (1493, 4.7%)
Neuroscience (971, 3.1%)	Food Insecurity (1377, 4.4%)
Obstetrics & Gynecology (947, 3.0%)	Olanzapine (902, 2.9%)
Psychiatry & Psychology (825, 2.6%)	Sarcopenia (888, 2.8%)
Neurodegenerative Diseases (818, 2.6%)	Gut Microbiota (633, 2.0%)
Substance Abuse (794, 2.5%)	Dementia (615, 1.9%)
Inflammatory Bowel Diseases & Infections (731, 2.3%)	Docosahexaenoic Acid (602, 1.9%)
Lipids (684, 2.2%)	Physical Activity (582, 1.8%)
Vitamin Metabolism (670, 2.1%)	Postpartum Depression (491, 1.6%)

**Table 6:** Top 10 most cited publications on nutrition and mental health.

Publication Title	First Author, Journal, Year	Number of Citations
Disability-adjusted life years (DALYs) for 291 diseases and injuries in 21 regions, 1990–2010: a systematic analysis for the Global Burden of Disease Study 2010 [5]	Murray, CJL et al., *Lancet*, 2012	6101
Global, regional, and national incidence, prevalence, and years lived with disability for 310 diseases and injuries, 1990–2015: a systematic analysis for the Global Burden of Disease Study 2015 [6]	Vos, T et al., *Lancet*, 2016	2797
The Microbiota-Gut-Brain Axis [7]	Cryan, JF et al., *Physiological Review*, 2019	2498
Maternal and child undernutrition: consequences for adult health and human capital [8]	Victora, CG et al., *Lancet*, 2008	2442
Mortality, morbidity, and risk factors in China and its provinces, 1990–2017: a systematic analysis for the Global Burden of Disease Study 2017 [9]	Zhou, MG et al., *Lancet*, 2019	2331
No health without mental health [10]	Prince, M et al., *Lancet*, 2007	2215
Developmental potential in the first 5 years for children in developing countries [11]	Grantham-McGregor, S, et al., *Lancet*, 2007	2087
Heart Disease and Stroke Statistics-2023 Update: A Report from the American Heart Association [12]	Tsao, CW, et al., *Circulation*, 2023	1839
Comparative efficacy and tolerability of 15 antipsychotic drugs in schizophrenia: a multiple-treatments meta-analysis [13]	Leucht, S, et al., *Lancet*, 2013	1825
Physical illness in patients with severe mental disorders. I. Prevalence, impact of medications and disparities in health care [14]	De Hert, M et al., *World Psychiatry*, 2011	1639

**Table 7:** Top 5 most cited publications on RCTs about nutrition and mental health.

Publication Title	First Author, Journal, Year	Number of Citations
Rapid and sustained symptom reduction following psilocybin treatment for anxiety and depression in patients with life-threatening cancer: a randomized controlled trial [15]	Ross, S, et al., *Journal of Psychopharmacology*, 2016	954
Maternal supplementation with very-long-chain *n*-3 fatty acids during pregnancy and lactation augments children’s IQ at 4 years of age [16]	Helland, IB, et al., *Pediatrics*, 2003	668
Cognitive behavior therapy-based intervention by community health workers for mothers with depression and their infants in rural Pakistan: a cluster-randomised controlled trial [17]	Rahman, A, et al., *Lancet*, 2008	631
ω-3 fatty acid treatment in 174 patients with mild to moderate Alzheimer disease: OmegAD study: A randomized double-blind trial [18]	Freund-Levi, Y, et al., *Archives of Neurology*, 2006	611
Mediterranean Diet and Age-Related Cognitive Decline A Randomized Clinical Trial [19]	Valls-Pedret, C, et al., *JAMA Internal Medicine*, 2015	599

## Data Availability

The original contributions presented in this study are included in the article. Further inquiries can be directed to the corresponding author.

## Supplementary Materials

The following supporting information can be downloaded at: https://www.mdpi.com/article/10.3390/nu17030399/s1, File S1: Keywords included in the five or seven clusters for all articles published on nutrition and mental health in 2014 and 2024, respectively.
