# Supplementary material for: Mapping the Scientific Research on Nutrition and Mental Health: A Bibliometric Analysis
Source: Nutrients. 2025 Jan 22;17(3):399. doi: 10.3390/nu17030399 (PMC11819674; doi:10.3390/nu17030399)
Supplement: Supplementary file 1 [file nutrients-17-00399-s001.zip › nutrients-3413114-supplementary.pdf]

**2024**

**cluster 1: public health & social factors (53 items, red)**

- accessibility
- adolescents
- barriers
- burden
- care
- children
- climate change
- consequences
- covid-19
- determinant
- disparitites
- distress
- education
- experiences
- exposure
- fatigue
- food insecurity
- gender
- growth
- health
- health equity
- hiv/aids
- impact
- income
- intervention
- life-style intervention
- low-income
- mental-health
- national-health
- outcomes
- pandemic
- people
- physical health
- poverty
- primary-care
- program
- psychological distress
- public health
- qualitative methods
- quality-of-life
- randomized controlled trial
- resilience
- security
- self-efficacy
- social determintant
- social support
- stigma
- substance use
- support
- survivors
- trends
- women
- young-adult

**cluster 2: interventions and biological mechanisms (51 items, green)**

- acid
- antidepressant drugs
- antioxidant
- antipsychotic-drugs
- anxiety disorder
- bipolar disorder
- body-weight gain
- brain
- consumption
- depression
- dietary fiber
- dopamine
- double-blind
- efficacy
- expression
- fatty-acids
- gut-brain-axis
- illness
- inflammation
- ketogenic diet
- lsd / lysergic-acid diethylamid
- major depression
- mechanisms
- memory
- mental-illness
- metaanalysis
- metabolic syndrome
- metabolism
- microbiota

- model
- mood
- omega-3-fatty-acids
- oxidative stress
- pain
- polyunsaturated fatty-acids
- posttraumatic-stress-disorder
- prebiotics
- probiotics
- psilocybin use
- psychedelics
- psychiatric-disorders
- psychiatry
- psychosis
- rats
- schizophrenia spectrum disorder
- serotonin
- supplementation
- systematic review
- therapy

**cluster 3: health of the elderly**
**(32 items, yellow)**

- adults
- age
- aging
- alzheimer-disease
- association
- blood-pressure
- body-composition
- cancer
- cardiovascular health
- china
- cognition
- cognitve function
- cognitive impairment
- community
- decline
- dementia
- elderly
- frailty
- healhty lifestyle
- impairment
- malnutrition
- mini-mental-state
- mortality
- nutritional-status
- older-adults
- performance
- population
- predictors
- prevention
- risk
- risk-factor
- sarcopenia

**cluster 4: lifestyle behavior**
**(41 items, blue)**

- adherence
- alcohol
- alcohol-consumption
- behavior-change
- college-students
- depressive symptoms
- diet
- dietary
- dietary quality
- exercise
- food
- generalized anxiety disorder
- health behaviours
- health promotion
- healthy diet
- index
- knowledge
- life
- life-style
- life-style behaviors
- mediterranean diet
- men
- nutrition
- patterns
- physical-activity
- psychometric properties
- quality
- questionnarie
- recommendations

- reliability
- satisfaction
- scale
- sedentary behavor
- sleep
- sleep quality
- smoking
- stress
- students
- university-students
- validity
- well-being

**cluster 5: nutrition-related illness (30 items, purple)**

- anorexia-nervosa
- associations
- attitudes
- bariatric surgery
- behavior
- binge-eating disorder
- body-image
- body-mass-index
- body-weight
- bulimia-nervosa
- cardiometabolic health
- co-morbidity
- diabetes-mellitus
- diagnosis
- eating behavior
- eating-disorder
- epidemiology
- follow-up
- food addiction
- food-intake
- guideline
- insulin-resistance
- management
- obesity
- overweight
- prevalcence
- sample
- treatment
- validation
- weight-loss

**cluster 6: observational studies (5 items, petrol)**

- cross-sectional study
- NHANES
- PHQ-9
- severity
- symptoms

**cluster 7: pregnancy and postpartum period (4 items, orange)**

- dietary patterns
- postpartum depression
- postpartum period
- pregnancy

**2014**

**cluster 1: public health & social factors (30 items, red)**

- adults
- care
- community
- depressive symptomatology
- depressive-disorders
- diabetes
- education
- food insecuirty
- health
- hiv/aids
- hunger
- impact
- insufficiency
- intervention
- management
- mental-health
- metaanalysis
- outcomes
- poverty
- prevention
- program
- quality-of-life
- questionnaire
- reliability
- scale
- social support
- symptoms
- therapy
- validation
- validity

**cluster 2: interventions and biological mechanisms (47 items, green)**

- addiction
- age
- antidepressant drugs
- antioxidant
- anxiety
- attention-deficit hyperactivity disorder
- autism
- bipolar affective-disorder
- brain
- central-nervous-sysetem
- children
- dietary patterns
- double-blind
- expression
- fatty-acids
- food
- growth
- gut microbiota
- illness
- infants
- inflammation
- insulin-resistance
- life
- major depression
- memory
- mental-retardation
- metabolism
- mood
- nutrition
- omega-3-fatty-acids
- oxidative stress
- performance
- placebo-controlled trial
- plasma
- polyunsaturated fatty-acids
- potpartum depression
- pregnancy
- psychiatric-disorder
- psychological distress
- quality
- randomized controlled-trial
- school
- sleep
- smoking
- stress
- supplementation

**cluster 3: health of the elderly**
**(21 items, yellow)**

- adherence
- alzheimer-disease
- assocaition
- cancer
- cognitive impairment
- consumption
- decline
- dementia
- disability
- elderly
- epidemiology
- health-care
- impairment
- malnutrition
- mediterranean diet
- mini-mental-state
- nutritional -status
- older-adults
- population
- predictors
- risk

**cluster 4: lifestyle behavior**
**(21 items, blue)**

- antipsychotic-drugs
- cardiovascular-disease
- co-morbidity
- coronary-heart disease
- diet
- exercise
- health behavior
- life-style
- mental-disorders
- metabolic syndrome
- mortality
- nutrition examination survey
- pattern
- people
- physical healht
- physical-activity
- posttraumatic-stress
- primary-care
- risk-factor
- schizophrenia
- weight-gain

**cluster 5: nutrition-related illness**
**(28 items, purple)**

- adolescents
- anorexia-nervosa
- associations
- attitudes
- behavior
- binge etaing
- binge-eating disorder
- body-composition
- body-mass-index
- body-weight
- bulimia-nervosa
- college-students
- diagnosis
- eating-disorder
- efficacy
- follow-up
- food addiction
- food-intake
- gender
- mental-illness
- obesity
- overweight
- prevalence
- treatment
- weight
- weight-loss
- women
- young-adults
